# *Tetracapsuloides bryosalmonae* infection affects the expression of genes involved in cellular signal transduction and iron metabolism in the kidney of the brown trout *Salmo trutta*

**DOI:** 10.1007/s00436-015-4425-z

**Published:** 2015-03-20

**Authors:** Gokhlesh Kumar, Subhodeep Sarker, Simon Menanteau-Ledouble, Mansour El-Matbouli

**Affiliations:** Clinical Division of Fish Medicine, Department for Farm Animals and Veterinary Public Health, University of Veterinary Medicine, Veterinärplatz 1, 1210 Vienna, Austria

**Keywords:** Salmonids, Myxozoan parasite, Proliferative kidney disease, Intraluminal sporogonic stages, Gene expression

## Abstract

*Tetracapsuloides bryosalmonae* is an enigmatic endoparasite which causes proliferative kidney disease in various species of salmonids in Europe and North America. The life cycle of the European strain of *T. bryosalmonae* generally completes in an invertebrate host freshwater bryozoan and vertebrate host brown trout (*Salmo trutta*) Linnaeus, 1758. Little is known about the gene expression in the kidney of brown trout during the developmental stages of *T. bryosalmonae*. In the present study, quantitative real-time PCR was applied to quantify the target genes of interest in the kidney of brown trout at different time points of *T. bryosalmonae* development. PCR primers specific for target genes were designed and optimized, and their gene expression levels were quantified in the cDNA kidney samples using SYBR Green Supermix. Expression of Rab GDP dissociation inhibitor beta, integral membrane protein 2B, NADH dehydrogenase 1 beta subcomplex subunit 6, and 26S protease regulatory subunit S10B were upregulated significantly in infected brown trout, while the expression of the ferritin M middle subunit was downregulated significantly. These results suggest that host genes involved in cellular signal transduction, proteasomal activities, including membrane transporters and cellular iron storage, are differentially upregulated or downregulated in the kidney of brown trout during parasite development. The gene expression pattern of infected renal tissue may support the development of intraluminal sporogonic stages of *T. bryosalmonae* in the renal tubular lumen of brown trout which may facilitate the release of viable parasite spores to transmit to the invertebrate host bryozoan.

## Introduction

Proliferative kidney disease (PKD) is an economically important disease affecting various species of salmonids in Europe and North America (Hedrick et al*.* 1993; El-Matbouli and Hoffman 1994). The causative agent of PKD is *Tetracapsuloides bryosalmonae* (Anderson et al*.* 1999; Canning et al*.* 1999; Feist et al. 2001) which belongs to the phylum Myxozoa and class Malacosporea. The *T. bryosalmonae* life cycle alternates between an invertebrate host, bryozoan, and a vertebrate host, salmonids (Canning et al. 1999, 2002; Okamura et al. 2011). PKD has been implicated in the decline of wild brown trout (*Salmo trutta*) Linnaeus, 1758 and other wild populations of salmonids especially, in Switzerland, Norway, and Estonia (Wahli et al. 2002; Sterud et al. 2007; Dash and Vasemägi 2014). Spores of *T. bryosalmonae* develop in the kidney tubules of infected brown trout and are released via urine to infect freshwater bryozoans (Morris and Adams 2006; Grabner and El-Matbouli 2008). Low numbers of interstitial presporogonic stages of *T. bryosalmonae* were observed in the kidney samples of brown trout, and high numbers of intraluminal sporogonic stages of *T. bryosalmonae* were seen at 8–12 weeks post-exposure (wpe), but it was almost nil at 6 wpe (Kumar et al. 2013). It has been recently demonstrated that *T. bryosalmonae* is able to persist in chronically infected brown trout and could to infect the bryozoan, *Fredericella sultana* colonies up to 104 wpe (Abd-Elfattah et al. 2014).

Knowledge about gene expression can be used to understand how the parasite affects host renal cell activities and mechanisms during the developmental stages toward explaining pathogenesis of the parasite in targeted tissue. Some studies have examined immune-related gene expression in the kidney of rainbow trout (*Oncorhynchus mykiss*) Walbaum, 1792 naturally infected with *T. bryosalmonae* (Holland et al. 2003; Gorgoglione et al. 2013; Kumar et al. 2015). However, very little is known about gene expression in brown trout; only few genes involved in cellular stress, growth, hemoglobin, and calcium metabolism have been examined in the kidney of brown trout during *T. bryosalmonae* developmental stages (Kumar et al. 2014).

A number of genes of interest were selected based on our previous study, where we compared transcriptomes from the kidneys of infected and noninfected brown trout by suppressive subtractive hybridization and discovered transcripts in the kidneys of brown trout (Kumar et al. 2014). Afterward, the relative gene expression of these genes in the kidney of infected brown trout was examined at different time points of *T. bryosalmonae* development. New results on the expression of brown trout genes would offer insights on the modulation of renal tissue signal transduction, proteasomal activities, including membrane transporters, as well as the role of proteins involved in cellular iron storage in gene regulatory mechanisms in brown trout during parasite development.

## Materials and methods

### Fish samples

Infected kidney samples were originated from our previous study (Kumar et al. 2014). Briefly, 60 brown trout were infected with the spores of *T. bryosalmonae*, released from mature sacs of parasite from laboratory-infected *Fredericella sultana* colonies and maintained at 16.5 ± 1 °C during the whole experimental period. Additional 30 brown trout were held as a noninfected fish. Posterior kidneys were sampled from both infected (*n* = 10) and noninfected groups (*n* = 5) at different time points such as 6, 8, 10, and 12 wpe. Infection in individual kidney sample was first confirmed by quantitative real-time PCR (qPCR) of *T. bryosalmonae* according to the method described by Grabner and El-Matbouli (2009). At 6 wpe, parasite load was low, while at 8–12 wpe, parasite load was high in the kidney of infected brown trout (Kumar et al. 2013). Additionally, *T. bryosalmonae* stages were determined in the kidney samples using anti-*T. bryosalmonae* monoclonal antibody (Aquatic Diagnostics), and antibody–antigen reaction was visualized using a Dako EnVision+ System-HRP (AEC) kit.

### Gene selection

Based on work previously conducted in our laboratory (Kumar et al. 2014), where we identified transcripts in the kidney of brown trout, which exhibited signal transduction, ion transporter, and cellular iron activities, based on this, we selected six genes of interest in the present study: Rab GDP dissociation inhibitor beta, gamma-secretase subunit PEN-2, integral membrane protein 2B (ITM2B), NADH dehydrogenase 1 beta subcomplex subunit 6 (NDUFB6), 26S protease regulatory subunit S10B (PSMC6), and ferritin middle M subunit.

### Quantitative real-time PCR 

**Fig. 1:**
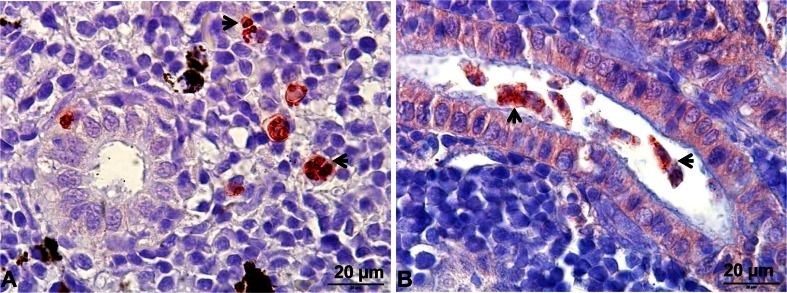
*Tetracapsuloides bryosalmonae* stages in the kidney of brown trout. **a** Interstitial presporogonic parasite stages (*arrows*), **b** intraluminal sporogonic parasite stages (*arrows*) in the renal tubule. Parasite stages were visualized by immunohistochemistry using anti-*T. bryosalmonae* monoclonal antibody IgG1 isotype P01 and counterstained with hematoxylin

Total RNA was extracted from the selected kidney samples collected at interstitial presporogonic (6 wpe) (Fig. 1a) and high numbers of intraluminal sporogonic stages of parasite (Fig. 1b) (8, 10, and 12 wpe) with low numbers of interstitial presporogonic parasite stages and from non-infected brown trout, using an RNeasy Mini Kit (Qiagen) according to the manufacturer’s instructions, and included an on-column DNase digestion step. cDNA was synthesized using an iScript cDNA Synthesis Kit (BIO-RAD) with 1 μg total RNA per the user’s manual.

PCR primers specific for the 6 selected target genes were designed (Table 1). PCR assays were optimized using gradient PCRs to determine the optimal annealing temperature and primer concentration. qPCR reactions had a final volume of 20 μl and contained 4 μl of 1:10-fold diluted cDNA, 0.4 μM of each primer, 1X SYBR Green Supermix (Bio-Rad), and DEPC-treated sterile distilled water. After 5 min of cDNA denaturation at 95 °C, 38 cycles were performed at 95 °C for 30 s, 57 or 62 °C for 30 s, and 72 °C for 30 s. A melting-point curve was then measured, starting from 57 or 62 °C and increasing by 0.5 °C every 10 s up to 95 °C, to detect any nonspecific PCR products. Each qPCR was performed in triplicate. Elongation factor alpha 1 (Kumar et al. 2014) and beta-actin (Rucker and El-Matbouli 2007) were used as reference genes for normalization of target genes. Standard curves were constructed for the target and reference genes to measure the quantity of target genes in the kidney samples. Relative expression levels of the target genes were analyzed at each time point using a linear mixed effect model. Adjustment for multiple comparisons was performed using SIDAK’s procedure. The differences between groups (infected and noninfected) at each single time point were analyzed using *t* tests for independent samples with Bonferroni α-correction. Correlations between relative expression levels were analyzed by calculating the Pearson product-moment correlation coefficient. For all statistical tests, a *p* value <0.05 was regarded as significant.

**Table 1:** Nucleotide sequence of quantitative real-time PCR primers used in this study

Primer name	Sequence (5′–3′)	Annealing temperature (°C)	Amplicon size (bp)	GeneBank accession no.
Rab GDI F	CTGCGACGACATCAAGGACA	62	133	JZ713062
Rab GDI R	AGTGTTGCAGACTCGCTCAT			
PEN-2 F	GCTGTCAATACGAAGGGGGA	62	128	JZ713064
PEN-2 R	GCCACAGGAACGGAAGGAAT			
Integral membrane F	GACGTGCTAAACACACTCGCT	62	138	JZ713055
Integral membrane R	CCTTGTCCTCGGGAATTAGGG			
NADH F	GACTGGTTACACAGCAGACGA	62	145	JZ713060
NADH R	GCCAGCTCAGAATTTTGCCA			
PSMC6 F	AAAGAGTTGAGGGAACAGCTCA	62	154	JZ713057
PSMC6 R	GAGGTCCATTGGTTGCCTTG			
Ferritin F	CCGACAAGCTACTCTCCTTCC	62	200	JZ713059
Ferritin R	GAAGTCACACAGATGGGGGT			
EF-1α F	AGACAGCAAAAACGACCCCC	57	167	HF563594
EF-1α R	AACGACGGTCGATCTTCTCC			
Beta-actin F	ATGGAAGGTGAAATCGCC	53	260	AF157514
Beta-actin R	TGCCAGATCTTCTCCATG			

## Results

Rab GDI beta, ITM2B, NDUFB6, PSMC6, and ferritin M genes were differentially expressed in the kidney of brown trout during parasite development. The expression of these genes (Rab GDI, ITM2B, NDUFB6, and PSMC6) exhibited a significant positive correlation between each other. The correlation of these target genes was analyzed with the parasite load at different time points. For this, relative gene expression values of *T. bryosalmonae* were used from our previous study, where 18S rDNA gene of *T. bryosalmonae* was quantified in the infected kidney of brown trout at different time points (Kumar et al. 2013). We found that the gene expression level of Rab GDI, PEN-2, and PSMC6 exhibits nonsignificant positive correlation (*r* = 0.426 and *p* = 0.163; *r* = 0.0609 and *p* = 0.850; *r* = 0.287 and *p* = 0.362) with the parasite load; however, ITM2B and NDUFB6 exhibit a significant positive correlation (*r* = 0.762 and *p* = 0.003; *r* = 0.571 and *p* = 0.045) with the parasite load.

**Fig. 2:**
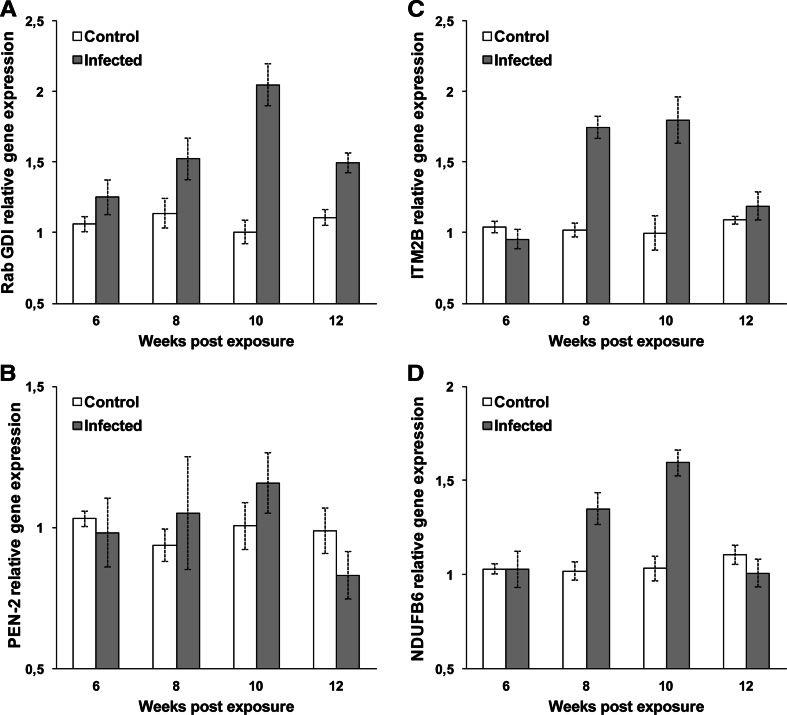
Quantitative real-time PCR showing relative expression profiles of selected genes in infected and noninfected kidney of brown trout. Relative gene expression changes were determined by calculating the mean expression values from the infected and noninfected kidney samples. Each value represents the mean of three independent biological samples and *error bars* indicate standard deviation **a** Rab GDP dissociation inhibitor beta, **b** Gamma-secretase subunit PEN-2, **c** integral membrane protein 2B, **d** NADH dehydrogenase 1 beta subcomplex subunit 6

Expression of Rab GDI was significantly upregulated (*p* < 0.016 or *p* < 0.003) in infected brown trout at 8–12 wpe (Fig. 2a). Expression of PEN-2 was not significantly upregulated (*p* = 0.593 or *p* = 0.093) at any of the tested time points in brown trout (Fig. 2b). Expression of both ITM2B and NDUFB6 was significantly upregulated (*p* < 0.022 or *p* < 0.024) in infected brown trout at 8–10 wpe but not at 6 and 12 wpe (Fig. 2c, d).

**Fig. 3:**
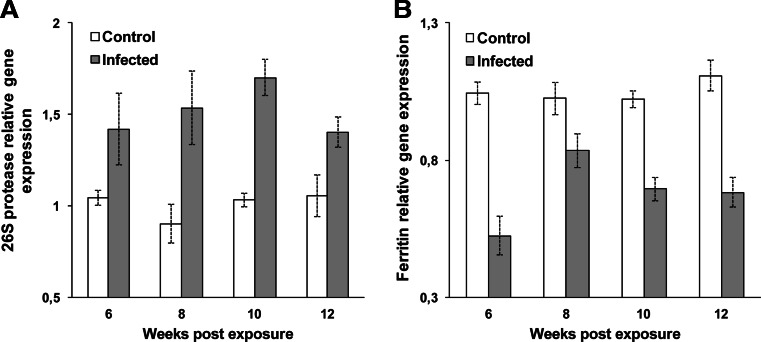
Quantitative real-time PCR showing relative expression profiles of selected genes in infected and noninfected kidney of brown trout. Relative gene expression details as in Fig. 2. **a** 26S protease regulatory subunit S10B, **b** ferritin middle subunit

Expression of PSMC6 was significantly upregulated at all time points, 6–12 wpe (*p* < 0.031, *p* < 0.014, *p* < 0.003, and *p* < 0.003, respectively) in the kidney of infected brown trout (Fig. 3a). However, expression of ferritin M was significantly downregulated at all time points, 6–12 wpe (*p* < 0.0001, *p* < 0.008, *p* < 0.004, and *p* < 0.001, respectively) in infected brown trout (Fig. 3b) exhibiting significant negative correlation with PSMC6 (*r* = −0.549 and *p* < 0.033).

## Discussion

Our understanding about gene expression in the kidney of brown trout during the developmental stages of parasite is limited. In the present study, we reported the gene expression study of six targeted genes in the kidney of brown trout at different time points during the developmental stages of the European strain of *T. bryosalmonae*. We observed significant transcriptional upregulation and downregulation of these targeted host gene responses in the kidney of brown trout at different time points of parasite infection. The highest gene expression of Rab GDI, ITM2B, NDUFB6, and PSMC6 genes is observed in the time period of 8–10 wpe of *T. bryosalmonae*, where the highest number of intraluminal sporogonic stages and high parasite load occurred in the infected kidney of brown trout (Kumar et al. 2013). This data support our previous study that the expression levels of host genes such as cold-inducible RNA-binding protein, cyclin-dependent kinase inhibitor 2A, transforming protein RhoA, immunoglobulin light chain, and major histocompatibility complex are highly upregulated during intraluminal sporogonic stages and high parasite load in the kidney of brown trout (Kumar et al. 2014), suggesting that they may play an important role in the development of parasite sporogonic stages in the kidney of brown trout.

The Rab guanosine diphosphate-dissociation inhibitor protein 2 regulates the GDP/GTP exchange reaction of most Rab proteins by inhibiting their dissociation and subsequent binding by GDP (Sedlacek et al. 1999). Information about Rab GDI isoforms and their differential expression in fish during the infection of microorganisms is limited. However, Rab1 has been isolated in red drum (*Sciaenops ocellatus*), and its expression has been detected in a number of organs such as the kidney and spleen during bacterial infection (Hu et al. 2011). It has been implicated in the disease development in humans as well as aquatic animals (Stone et al. 2007; Hu et al. 2011). In the present study, it was found that expression of Rab GDI was upregulated significantly in brown trout at all the time points of parasite development. Other signal transduction regulatory proteins such as transforming protein RhoA are upregulated in the kidney of brown trout in response to *T. bryosalmonae* infection at 8–10 wpe (Kumar et al. 2014). This suggests that upregulation of protein transport, membrane recycling, and mediating trafficking may facilitate in the development of parasite sporogony in the kidney of brown trout.

The ITM2B are a family of proteins which comprises amyloid precursor proteins which are processed by the beta-secretase and gamma-secretase complexes to yield beta-amyloid peptides (Kim et al. 2008). Complete mRNA coding sequences of ITM2B have been identified in organs of Atlantic salmon (*Salmo salar*) (Leong et al. 2010); however, the involvement of this gene in the disease process in fish species have yet to been investigated. In this study, we first examine the upregulation of ITM2B in infected brown trout during *T. bryosalmonae* development at 8–10 wpe which is characterized by the presence of numerous sporogonic stages of the parasite in the kidney lumen of infected brown trout; these results suggest that transmembrane proteins may have an important role in the migration of *T. bryosalmonae* to the renal lumen. Interestingly, in p53^+/+^, as well as p53^−/−^ cell lines, ITM2B gene expression is able to induce apoptotic-cell death, suggesting a p53-independent apoptotic role of ITM2Bs (Fleischer and Rebollo 2004). This contrasts with PEN-2 which has been implicated in p53-dependent apoptosis in zebrafish (*Danio rerio*), as discussed later and suggests that *T. bryosalmonae* might be able to induce apoptosis in the host cells through several independent mechanisms (Campbell et al. 2006; Fleischer and Rebollo 2004).

Mitochondrial membrane enzyme such as NADH dehydrogenase 1 has been upregulated in brown trout by *T. bryosalmonae* infection. NADH dehydrogenase 1 beta subcomplex subunit 6 is located in the inner mitochondrial membrane and constitutes one of the entry enzymes of oxidative phosphorylation in the mitochondria (Clason et al. 2010). NADH dehydrogenase alpha subcomplex is upregulated in the head kidney of Atlantic salmon during the course of infectious salmon anemia (LeBlanc et al. 2010). EcGRIM-19 is a nuclear encoded subunit of complex I that shows similarity to the NADH dehydrogenase 1 alpha subcomplex 13 and has been functionally characterized in the orange-spotted grouper (*Epinephelus coioides*). It has been reported that EcGRIM-19 induces apoptotic cell death in the organs of orange-spotted grouper in response to lipopolysaccharide of *Escherichia coli* (Shi et al. 2013). We found that the expression of NDUFB6 was significantly upregulated in the kidney of brown trout during developmental stages of *T. bryosalmonae.* Given the dearth of information regarding NDUFB6 in salmonids, it would be of considerable interest to study mitochondrial oxidative phosphorylation in the brown trout during parasite development and to investigate whether mitochondrial membrane enzymes are in anyway involved in renal tissue apoptosis during parasite development. This aspect is currently being investigated.

The 26S protease regulatory subunit S10B was upregulated during infection of parasite in brown trout. PSMC6 is involved in the ATP-dependent intracellular degradation of ubiquitinated proteins (Hershko and Ciechanover 1998) in particular following cellular stress. Proteosome 26S subunits are differentially upregulated in the skin mucus of Atlantic cod (*Gadus morhua*) and epithelioma papulosum cyprini cells of fathead minnow (*Pimephales promelas*) in response to *Vibrio anguillarum* and spring viremia of carp, respectively (Rajan et al. 2013; Liu et al. 2013). In the current study, 26S proteasome levels were significantly higher in the kidney of infected brown trout at all time points of *T. bryosalmonae* development. This data suggests that PSMC6 was upregulated in an attempt to respond to the stress inflected on the host cells by the infection.

Gamma-secretase subunit PEN-2, or Presenilin-2, is an essential regulatory component of the gamma-secretase complex, which is a protease complex responsible for the proteolysis of transmembrane proteins such as Notch and the amyloid precursor protein, implicated in human diseases such as Alzheimer’s disease (Francis et al. 2002). In zebrafish, low levels of PEN-2 have been correlated to p53-dependent apoptosis contributing to neuronal loss. This suggests that PEN-2 plays an important role in promoting neuronal cell survival and protection from apoptosis in vivo (Campbell et al. 2006). We found that the expression of PEN-2 was not significantly upregulated at any time points in infected brown trout; however, other translation and protein biosynthesis molecule such as ribosomal protein L6 are significantly upregulated in the infected kidney of brown trout (Kumar et al. 2014).

Ferritin is an intracellular protein complex which stores iron in a soluble, nontoxic, readily available form. It is important for iron homeostasis as it shows ferroxidase activity (Elvitigala et al. 2013) and involved in antibacterial activities, immune response, and iron metabolism of different fish species (Neves et al. 2009; Zheng et al. 2010; Wang et al. 2011). In other teleosts such as the rock bream (*Oplegnathus fasciatus*), exposure to microbial pathogens and pathogen-derived mitogens, ferritin H-like subunit expression was markedly elevated in the blood stream (Elvitigala et al. 2013, 2014). The present data showed that the expression of ferritin M subunit was significantly downregulated at all time points in infected brown trout, which exhibited negative correlation (*r* = −0.015) with parasite load. Similarly, hemoglobin gene carries an iron-containing molecule is downregulated in the kidney of brown trout during the *T. bryosalmonae* development (Kumar et al. 2014). This suggests that the infection of *T. bryosalmonae* interfered with the iron metabolism and immune response of its host.

A hallmark of several metabolic diseases is protein misfolding (Kim et al. 2008). For example, ITM2B and PEN-2 are involved in the pathophysiology of Alzheimer’s disease in *Homo sapiens* characterized by plaque formation (Kim et al. 2008). Similarly, PSMC6 is a member of the proteasome complex that is involved in the degradation of misfolded protein. The Rab family of proteins is central to the effective intracellular transport of integral membrane proteins such as ITM2B or PEN-2, including secretable soluble proteins via vesicle-mediated mechanisms (Sedlacek et al. 1999; Pfeffer 2012). These would also include transport of neurostransmitter molecules in transporter vesicles which upon membrane fusion would lead to extracellular neurotransmitter release in response to appropriate extracellular stimuli or cues (Südhof and Rizo 2011). This may explain the similarity in the gene expression pattern of Rab GDP-dissociation inhibitor beta with ITM2B postinfection with *T. bryosalmonae*. The highest gene expression is observed in the time period of 8–10 wpe. It may be that impairment in the function of Rab GDP-dissociation inhibitor correlates to an impairment in the intracellular trafficking of ITM2B in brown trout as a result of infection to *T. bryosalmonae*. On the other hand, the ferritin M subunit and the NDUFB6 are essential components of mitochondrial physiology and regulate oxidative phosphorylation. The gene expression of ferritin is in stark contrast to those of all other five genes. While the expression of NDUFB6 is consistent with the expressions of ITM2B, PSMC6, and Rab GDI, it is inconsistent with that of the ferritin M subunit.

Some of the genes such as ITM2B and NDUFB6 did not change significantly in the kidney samples after 10 wpe; however, the presence of parasite was confirmed by qPCR. Based on previously conducted studies (Kumar et al. 2013), it is known that *T. bryosalmonae* is multiplying exponentially in the tissues and that its numbers become constant between 10 and 12 wpe. It is therefore possible that this alteration in the NADH levels could be explained by the parasite’s aggressive multiplication and that, as its rate of multiplication decreases, so does its impact, until its effect on the gene expression stop being statistically significant. However, more research would need to be conducted to confirm this hypothesis. For example, it is of interest to investigate the roles of these two genes at time points beyond 12 wpe, especially in fish experiencing mortality due to the infection and contrasting these results to populations which survive beyond this time point with latent infection.

In conclusion, this study suggests that genes involved in cellular signal transduction and proteasomal activities, membrane transporters, and cellular iron storage protein of the brown trout are differentially upregulated or downregulated in the kidney of brown trout during parasite development, which may support the development of intraluminal sporogonic stages of *T. bryosalmonae* in the renal kidney tubular lumen of brown trout and the release of viable parasite spores via urine to transmit the invertebrate host bryozoan. This study provides key information to understand host renal tissue responses during the developmental stages of the parasite, and these host gene responses and associated signaling pathways may enable the development of effective intervention and therapeutic strategies targeting *T. bryosalmonae* in salmonids.
